# Wine, Polyphenols, and Mediterranean Diets. What Else Is There to Say?

**DOI:** 10.3390/molecules26185537

**Published:** 2021-09-12

**Authors:** Celestino Santos-Buelga, Susana González-Manzano, Ana M. González-Paramás

**Affiliations:** Grupo de Investigación en Polifenoles (GIP-USAL), Universidad de Salamanca, E-37007 Salamanca, Spain; susanagm@usal.es (S.G.-M.); paramas@usal.es (A.M.G.-P.)

**Keywords:** olive oil, resveratrol, alcohol, phytochemicals, tyrosol

## Abstract

A considerable amount of literature has been published claiming the cardiovascular benefits of moderate (red) wine drinking, which has been considered a distinguishing trait of the Mediterranean diet. Indeed, red wine contains relevant amounts of polyphenols, for which evidence of their biological activity and positive health effects are abundant; however, it is also well-known that alcohol, even at a low level of intake, may have severe consequences for health. Among others, it is directly related to a number of non-communicable diseases, like liver cirrhosis or diverse types of cancer. The IARC classifies alcohol as a Group 1 carcinogen, causally associated with the development of cancers of the upper digestive tract and liver, and, with sufficient evidence, can be positively associated with colorectum and female breast cancer. In these circumstances, it is tricky, if not irresponsible, to spread any message on the benefits of moderate wine drinking, about which no actual consensus exists. It should be further considered that other hallmarks of the Mediterranean diet are the richness in virgin olive oil, fruits, grains, and vegetables, which are also good sources of polyphenols and other phytochemicals, and lack the risks of wine. All of these aspects are reviewed in this article.

## 1. Introduction

In November 2010, following a transnational nomination submitted by Spain, Greece, Italy, and Morocco, the UNESCO decided to inscribe the Mediterranean Diet as an Intangible Cultural Heritage of Humanity (https://ich.unesco.org/en/Decisions/5.COM/6.41, accessed on 1 September 2021), further enlarged in December 2013 with the incorporation of three other countries: Croatia, Cyprus, and Portugal (https://ich.unesco.org/en/Decisions/8.COM/8.10, accessed on 1 September 2021). In its decision, the UNESCO recognized the Mediterranean diet (MedDiet) as “a set of skills, knowledge, practices and traditions ranging from the landscape to the table, including the crops, harvesting, fishing, conservation, processing, preparation and, particularly, consumption of food (…) characterized by a nutritional model that has remained constant over time and space, (…) always respecting beliefs of each community.”

Consistent associations of this Mediterranean dietary pattern with cardiovascular benefits were first reported in the 1960′s from the earlier results of the Seven Countries Study (https://www.sevencountriesstudy.com/, accessed on 1 September 2021), describing significantly lower mortality rates and incidences of cardiovascular diseases in the Italian, Greek, and Croatian cohorts than in the rest of the included (non-Mediterranean) countries [1]. Further confirmation of these outcomes was obtained from the HALE project (Healthy Ageing—a Longitudinal study in Europe), analysing data on lifestyle, dietary, and biological determinants of healthy ageing from individuals of 13 European countries, collected from the Seven Countries, as well as the FINE (Finland, Italy, Netherlands Elderly study) and SENECA (Survey in Europe on Nutrition in the Elderly—a Concerted Action) prospections. It was found that adherence to a MedDiet, together with a healthful lifestyle (i.e., being physically active, non-smoking for more than 15 years, and moderate alcohol intake) was associated with a more than 50% lower rate of all-causes and cause-specific mortality, including coronary heart disease (CHD), cardiovascular disease (CVD), and cancer [2].

Similar observations were made from many other epidemiological and intervention studies. A comprehensive analysis of the results from observational studies and randomised clinical trials—comprising a total population of over 12,800,000 individuals—was made by Dinu et al. [3], concluding that there was robust evidence to suggest that greater adherence to a Mediterranean diet style is associated with a reduced risk of overall mortality, CVD, overall cancer incidence, neurodegenerative diseases, and type-2 diabetes. In a previous screening across intervention trials, Serra-Majem et al. [4] also concluded that there was good evidence to suggest that a MedDiet improves the lipid profile, endothelial function, and blood pressure, despite the fact that the authors also highlighted that there were discrepancies on how the different studies defined and formulated the Mediterranean diet. 

**Figure 1:**
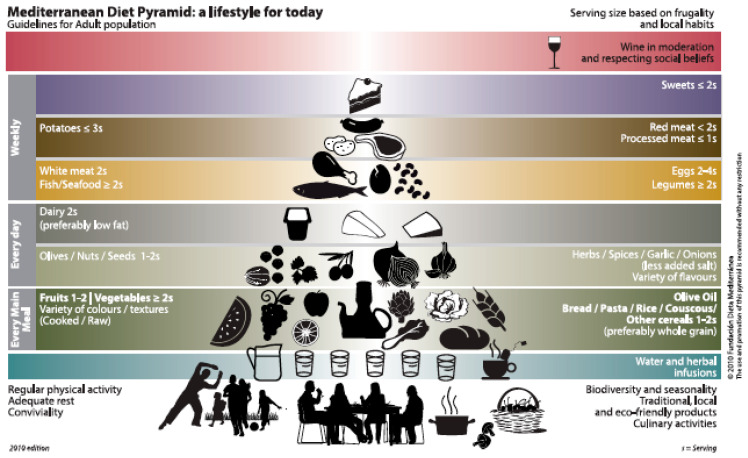
Pyramid of the Mediterranean diet (https://dietamediterranea.com/en/fundacion, accessed on 1 September 2021).

Indeed, the Mediterranean diet does not constitute a close and unique nutritional model, but it is rather a compendium of diverse dietary habits traditionally followed by countries around the Mediterranean basin. In spite of their heterogeneity, some common patterns are observed across these countries, namely a high consumption of plant products such as fruits, vegetables, legumes, and nuts, as well as cereals (bread, pasta, rice, and whole grains); a moderate intake of dairy products, fish, poultry, and eggs as main protein sources, with small amounts of red and processed meat; the use of olive oil as a main fat source and water as a beverage of choice. Additionally, the diet is characterized by infusions and optional moderate amounts of wine taken with meals, and a preference for seasonal, fresh, and locally low-processed products. This was summarized in the Pyramid of the Mediterranean diet proposed by the “Fundación Dieta Mediterránea” (https://dietamediterranea.com/en/fundacion, accessed on 1 September 2021) (Figure 1).

Relevant nutrients and bioactive compounds contributed by the main food items in the Mediterranean diet are summarized in Table 1.

The dietary patterns of the MedDiet have been suggested to likely overlap with those for optimal prevention of both cardiovascular diseases and cancer. Thus, it is considered that, whatever the particular food choices, Mediterranean diets provide adequate intakes of total fat and long-chain polyunsaturated fatty acids, dietary fiber, antioxidant vitamins, carotenoids, and polyphenols, as well as a balanced n-6/n-3 ratio of essential fatty acids and low amounts of saturated fatty acids (SFA) [5,6,7]. All of these characteristics are related to beneficial effects on endothelial and cardiovascular function. Furthermore, monounsaturated fatty acids (MUFA) present in olive oil (i.e., oleic acid) are acknowledged to improve the blood lipid profiles [8]. Actually, a common and consistent feature of the MedDiet seems to be the existence of a high MUFA/SFA ratio (estimated to be around 2.0 on average) [5], which has been significantly associated with low CVD mortality and overall mortality [9]. Complex carbohydrates and dietary fiber contributed by whole grain products, legumes, and vegetables have also been related with gut health and protection against different cancers, especially colorectal cancer [10,11]. There are also glucosinolates and other organosulphur compounds present in cruciferous vegetables and allium condiments, which have acknowledged anti-inflammatory properties [12]. Another feature of the MedDiet which is usually associated with its health-promoting properties is the supply of significant amounts of different classes of antioxidant polyphenols, which are even higher than those of other dietary antioxidants, such as vitamin C, vitamin E, or carotenoids [13]. Regular intake of these compounds has been related to beneficial effects on the lipids profile, blood pressure, glucose metabolism, adiposity, or inflammatory processes, and are also associated with a reduction in the incidence of several chronic diseases, like cardiovascular diseases, type-2 diabetes, metabolic syndrome, neurodegenerative disorders, or different cancers [14,15,16].

The purpose of the present review is to discuss the role of wine and wine polyphenols in the health benefits of the Mediterranean diet. A particular mention is made to resveratrol, owing to the special attention that has been paid to its possible contribution to the beneficial effects of moderate wine intake, as associated with the MetDiet. Reference is also made to olive oil as a distinguishing food in the MetDiet with claimed health benefits, which have been proposed to rely, at least in part, on its characteristic polyphenols, which are different to those present in wine.

## 2. Polyphenols as Key Components of Mediterranean Diets

**Figure 2:**
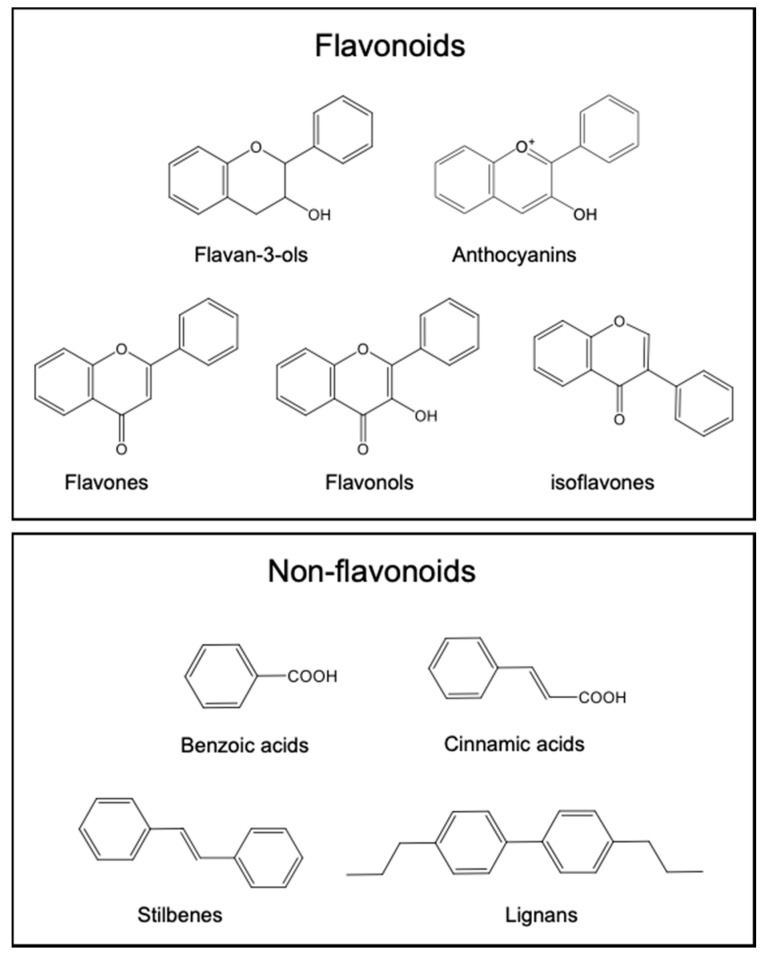
Core structures of the main classes of plant phenolic compounds.

Plant phenolic compounds, commonly referred to as polyphenols, are widespread in the diet, and are nowadays considered, at least in part, responsible for the health protective effects of fruit and vegetable-rich diets. They can be classified in two major classes: flavonoids and non-flavonoids, including phenolic acids (i.e., hydroxybenzoic and hydroxycinnamic acids and their derivatives), stilbenes, and lignans (Figure 2), as well as phenolic alcohols and their secoiridoid derivatives.

The dietary intake of polyphenols largely varies among individuals, and is estimated to range from a few hundred mg/day to more than 1800 mg/day depending on the region and target population, as well as the methodology used for the assessment [17]. Hydroxycinnamic acid esters, namely caffeoylquinic acids and flavan-3-ols oligo/polymers (i.e., proanthocyanidins), are usually reported as the most important groups of consumed polyphenols, followed by anthocyanins and flavonols [17]. In general, the contribution of phenolic acid derivatives and flavonoids tends to be equilibrated, although there are differences across countries and population groups as a function of their dietary habits. For example, there are higher proportions of flavonoids in Mediterranean regions, while phenolic acids would predominate in non-Mediterranean countries [18,19,20,21]. The main food sources for individual polyphenols tend to be similar among individuals, with coffee, tea, and fruits as major items, and vegetables and red wine in a second range [17]. It is suggested that moderate red wine drinkers consume polyphenols at levels well above the population average [6]. 

Despite the fact that the antioxidant capacity of polyphenols is well-substantiated in vitro and has been recurrently associated in the literature to their health effects, the little bioavailability and large biotransformation of most polyphenols in the organism raise doubts that this activity can have a primary role on their in vivo effects [17]. Although this possibility might not be discarded for particular compounds or situations, nowadays, other alternatives are considered to contribute to the in vivo effects of polyphenols. For example, they could act as modulators of gene expression and intracellular signaling cascades involved in cell function and protection [22,23]. There is also increasing evidence about the crucial role of the interactions between polyphenols and gut microbiota as a mainstay to explain the health benefits of their consumption. A vast majority of the consumed polyphenols reach the large intestine unaltered, where they can be catabolized by the colonic microflora to a variety of metabolites [24,25]. Some of these metabolites can be biologically active, and be responsible for the activity associated with their parent polyphenols. Among others, this would be the case of tyrosols, produced from oleuropein and related phenolics from olive oil, with putative effects against some types of cancer [26]; urolithins, involved in the lipid-lowering effects and improvement in the cardiovascular risk biomarkers of ellagitannins [27]; or estrogenic *S*-equol, enterodiol, and enterolactone, derived from soy isoflavones [28] and lignans [29], respectively. The role of other metabolites, such as phenolic acids and aldehydes resulting from the bacterial breakdown of flavonoids, is still uncertain, although they might be expected to contribute to a part of their effects, both at the local and systemic level. Additionally, unabsorbed polyphenols and phenolic metabolites can also have an impact on the composition of the gut microbiota, acting as prebiotic-like compounds. For instance, they have been suggested to be able to decrease the Firmicutes/Bacteroidetes ratio [30,31], linked to obesity trends in humans [32], and increase the abundance of beneficial *Bifidobacteria* and *Lactobacilli* spp. [30,33,34,35], while producing a reduction in the levels of Bacteroides, Streptococci, Enterobacteriacae, or Clostridia [36,37]. In the end, several mechanisms might be involved in the biological effects of polyphenols and contribute to their health benefits. This is a current active field of research that is rapidly progressing, so that advances are expected in coming years [38].

Most of the available information on the biological activity and effects of the phenolic compounds has been obtained from in vitro, ex vivo, and animal studies, whereas data directly obtained in humans are scarce, and restricted, in general, to short-term intervention trials on a reduced number of people. Some attempts have been made in Mediterranean cohorts, whose results support the role of polyphenol-rich foods to the health benefits of the Mediterranean diet [16,19,39,40]. Nevertheless, assessing the precise contribution of dietary polyphenols to those benefits remains complex, owing to the fact that the same food sources are also rich in other bioactives, such as vitamins, minerals, dietary fiber, or other antioxidants, which should also contribute to the health effects [17].

## 3. Olive Oil

Olive oil, and especially virgin olive oil (VOO), is one of the products most usually associated with the health properties of the MedDiet. Its regular consumption has been claimed to provide benefits against a number of disease conditions, such as atherosclerosis, diabetes mellitus, obesity, cancer, or neurodegenerative diseases [41]. It is well-known that olive oil is very rich in monounsaturated fatty acids—mainly oleic acid, accounting for up to 80% of its total fatty acids—with acknowledged positive effects on the profiles of plasmatic lipoproteins, triglycerides, and platelet aggregation [42,43], which has been linked to protection against cardiovascular and neurodegenerative diseases [44]. Actually, the EFSA has approved health claims regarding the positive effects of “monounsaturated fatty acids (mainly oleic acid)”, “oleic acid”, and “extra virgin olive oil” in the maintenance of normal blood LDL-cholesterol concentrations and the maintenance of normal (fasting) blood concentrations of triglycerides, when replacing saturated fatty acids (SFAs) in foods or diets [8].

In addition to its fatty acid profile, VOO also contains a series of biologically active polyphenols, in a concentration that oscillates within a large range from 50 to 1000 mg/kg, depending on the olive cultivar and ripening stage, environmental factors (climate, altitude, agricultural practices), extraction techniques, storage conditions, and time [45]. It has been estimated that they may account for up to around 2% of total olive oil weight, contributing not only to olive oil’s health properties, but also to its taste and fatty acid stability against oxidation [46].

**Figure 3:**
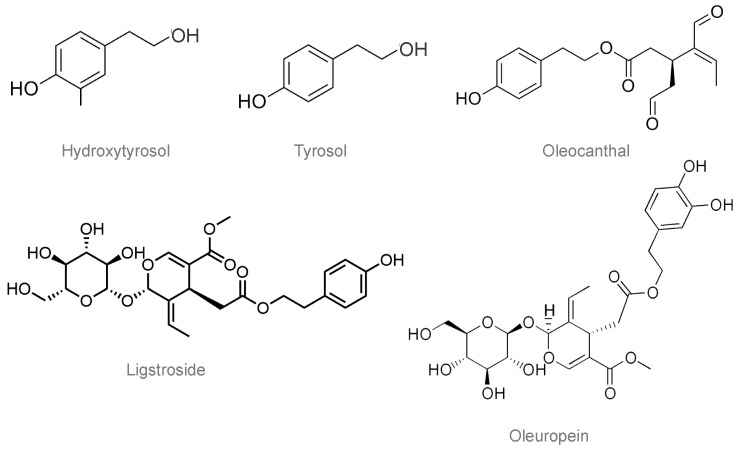
Representative polyphenols present in virgin olive oils.

VOO possesses a unique phenolic composition mainly consisting of secoiridoid derivatives, the most abundant one being oleuropein, the glucosylated form of 3,4-dihydroxyphenylethanol-elenolic acid (3,4-DHPEA-EA). Oleuropein is considered the main compound contributing to the bitterness of olives. Other related secoiridoids are the ligstroside aglycone (p-HPEA-EA) and the dialdehydic form of elenolic acid linked to either hydroxytyrosol (3,4-DHPEA-EDA; oleacein) or tyrosol (p-HPEA-EDA; oleocanthal), both existing as aglycones and glucosyl derivatives. Besides, VOO also contains phenolic alcohols such as tyrosol (p-HPEA) and hydroxytyrosol (3,4-DHPEA), mostly derived from their se-coiridoid precursors. The structures of these polyphenols are depicted in Figure 3. Other phenolic compounds also reported in lower amounts in VOO include lignans (pinoresinol, 1-acetoxypinoresinol, and 1-hydroxypinoresinol), verbascoside (i.e., caffeoylrhamnosyl-glucoside linked to hydroxytyrosol), some phenolic acids (vanillic, gallic, coumaric, caffeic acids) and flavonoids (especially flavonols derived from apigenin, luteolin, or quercetin) [46,47].

Olive oil phenolics have been extensively studied for their potential to counteract the onset and progression of a variety of chronic and aging-related diseases, and is attributed to hypoglycemic, anti-obesity, cardioprotective, neuroprotective, antimicrobial, and anti-cancer properties [47,48,49]. Several in vitro and in vivo studies have associated the health-promoting effects of olive oil phenolics to their antioxidant and anti-inflammatory potential as related to their ability to modulate a series of molecular pathways. Thus, they have been reported to be able to activate AMPK (AMP-activated protein kinase) with subsequent inhibition of the mTOR signaling pathway [22], which is involved in the regulation of adipose tissue functions, such as adipogenesis, thermogenesis, and lipid metabolism. It also modulates processes like mitochondrial biogenesis and functionality, hypoxia signaling, autophagy, and cell cycle progression [50]. In intervention studies, VOO-rich Mediterranean diets were deemed effective in reducing several inflammatory markers, such as C-reactive protein, TNF-α, interleukin-6 (IL6), endothelial adhesion molecules (VCAM-1, ICAM), or chemokines like MCP-1, which has been related to their polyphenol content [40,51]. A compound that demonstrated strong in vitro anti-inflammatory properties is oleocanthal, with a structure that resembles ibuprofen, which was shown to cause a dose-dependent inhibition of cyclooxygenase enzymes COX-1 and COX-2 [52]. Similarly, hydroxytyrosol was able to inhibit TNF-α, iNOS, and COX-2 in LPS-challenged human monocytic cell lines [53]. Additionally, in vitro and animal studies have reported that oleuropein and hydroxytyrosol may reduce fat tissue accumulation by downregulating the expression of adipogenesis-related genes like PGC-1α, lipoprotein lipase, acetyl CoA carboxylase-1, and carnitine palmitoyltransferase-1 [54]. Recent reviews can be consulted for further information on olive oil phenolic effects and mechanisms of action [44,47,49,55,56].

The beneficial effects of phenolic compounds from olives and olive oil (i.e., hydroxytyrosol and oleuropein complex) were recognized by the European Food Safety Authority (EFSA), which authorized health claims in relation to polyphenols in olives and the protection of LDL particles from oxidative damage, the maintenance of normal blood HDL-cholesterol concentrations, the maintenance of normal blood pressure, and “anti-inflammatory properties”. It also recognized their contribution to upper respiratory tract health, body defences against external agents, and the maintenance of a normally functioning gastrointestinal tract [57]. 

Within the PREDIMED study (http://www.predimed.es, accessed on 1 September 2021), a large Spanish trial on the primary prevention of chronic diseases through the Mediterranean Diet carried out in subjects at cardiovascular risk followed since 2013, it was estimated that olive oil and olives may provide about approximately 11% of the total polyphenol intake in a typical MedDiet, representing an important differential contribution to the profile of phenolic compounds consumed by Mediterranean populations [19]. Less optimistic calculations have been made by other authors. Thus, Parkinson and Cicerale [56], assuming a mean VOO intake of 30–50 g/day in Mediterranean countries, estimated that the amount of polyphenols ingested from VOO consumption would not exceed 9 mg/day. Whatever the dietary intake, at present there is not enough evidence to confirm that the consumption of olive phenolic compounds isolated by or as components of the VOO can be healthy [58]. Most of the in vivo studies with olive oil polyphenols have been carried out using supraphysiological concentrations that are difficult to extrapolate to a dietary context, while the number and variety of randomized clinical trials (RCT)—providing the highest level of scientific evidence—are very limited and insufficient to confirm their beneficial effects on humans, except for some markers of cardiovascular risk. Actually, the strongest piece of evidence has been obtained for the ability of VOO polyphenols to protect lipoproteins from oxidation and to reduce systolic blood pressure in hypertensive individuals [56]. Extensive RCT in different population groups with distinct disorders and at phenolic levels adjusted to usual VOO consumptions are, therefore, necessary to achieve high quality scientific evidence before nutritional recommendations can be given [56,58].

The health benefits attributed to olive oil could also be supposed for table olives. Nevertheless, the phenolic composition of table olives differs from that of olive oil, as they are influenced not only by the cultivar and harvesting time (green or fully ripened), but also by the processing conditions used for making them edible, which lead to chemical transformations in the polyphenols [59,60]. Thus, under the alkaline conditions used for the debittering of fruits in Spanish-style olives, oleuropein is hydrolyzed to practical disappearance. Moreover, in Greek-style black olives, in which the fruits are collected fully ripened and directly put into brine, an acid hydrolysis of oleuropein occurs, and orthodiphenols are oxidized and polymerized during the darkening step [60]. Tyrosol and hydroxytyrosol and their acetates have been identified as the most representative phenolic compounds in table olives, with concentrations of total polyphenols ranging between 200 mg/kg to 1200 mg/kg, depending on the cultivar and processing method, with oxidized olives containing the lowest levels. A further decrease of phenolic content is produced in pitted olives due to their loss in the washing liquids, which reduce their concentration to almost half that of the nonpitted fruits [60].

**Figure 4:**
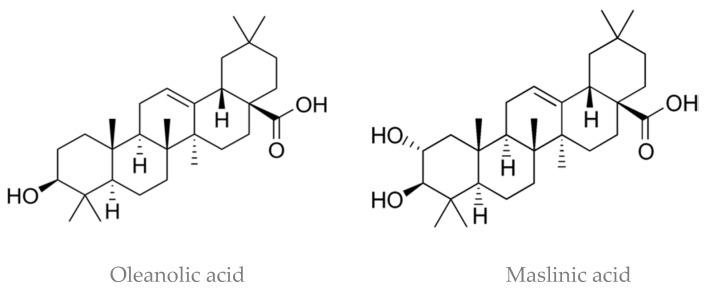
Structures of oleanolic and maslinic acids.

Besides polyphenols, olives also contain other bioactive compounds in the unsaponifiable fraction, such as pentacyclic triterpenoids like maslinic acid and oleanolic acid (Figure 4). A range of biological activities have been shown for maslinic acid, mostly from in vitro studies, such as anti-inflammatory, antiproliferative, antioxidant, and antidiabetic properties. In regard to oleanolic acid, hepatoprotective, antitumor, and antiviral properties have been reported [60,61,62]. These compounds are not lost during processing, and they are present in table olives and olive-pomace oil, a byproduct from olive oil extraction submitted to a refining process that leads to the complete loss of polyphenols. Table olives may contain more than 1300 mg/kg (dw) of maslinic acid, which is considered its richest food source [63]. Other dietary sources are spinach and eggplant, aromatic herbs, legumes, and to a lesser extent, some fruits like mandarin and pomegranate. Actually, plant-based diets including olives and olive oil, like the MedDiet, could provide a constant supply of maslinic acid, which might partly contribute to their health-enhancing properties [61].

## 4. Wine in the Context of the Mediterranean Diet

Wine is considered another distinguishing food of the Mediterranean diet contributing to its health benefits [44]. Nevertheless, it should not be forgotten that in several countries and regions that follow typical Mediterranean dietary patterns, alcohol, and therefore wine, is excluded for religious reasons.

Since the early St. Leger et al. [64] and Framingham studies [65], a lot of epidemiological evidence has accumulated, pointing to the existence of inverse relationships between light to moderate alcohol consumption—especially wine—and incidences and mortality of cardiovascular diseases (see, e.g., [66,67,68,69]), as well as of other chronic disorders like type 2 diabetes [70,71,72,73] or dementia and cognitive decline in old age [74,75]. The relationship has been described as a U- or J-shaped curve [76], with a minimum situated at a level of consumption around 10 to 30 g of alcohol/day. These studies are not free from debate, as they have been attributed to suffer from methodological limitations, which may have led to misinterpretations or biased conclusions [77,78,79,80]. Nevertheless, despite possible bias, many authors agree that when confounding factors are specifically adjusted, epidemiological trials still continue to be remarkably consistent regarding the beneficial effects from low to moderate alcohol/wine intake on CVD morbidity and mortality, as well as diabetes, osteoporosis, and neurological disorders [81,82,83].

A point of discussion is whether the purported wine benefits are due to ethanol or to other components. It is known that ethanol itself is able to increase HDL-cholesterol, prevent platelet aggregation, and enhance fibrinolysis, which may have positive effects on the cardiovascular system [84]. However, when differentiation among drinks is made, it is generally concluded that wine provides superior health benefits to other alcoholic drinks—especially spirits—either regarding protection against CVD [85,86,87,88], type 2 diabetes [73], or dementia [89]. This perception has also been supported by the results obtained in human clinical studies [90,91,92,93,94] and observations over Mediterranean cohorts [19,39,95].

The intended superior benefits of wine have been related to its phenolic compounds, which are absent or in very low concentrations in other alcoholic drinks. Wine contains a variable mixture of flavonoid and non-flavonoid compounds, extracted from the grape during winemaking. Phenolic contents in red wine is usually well above 1 g/L—concentrations that are higher than those that can be found in most fruits and vegetables—while in white wine, it does not commonly exceed a few hundred mg/L, due to the fact that it is not normally submitted to maceration with grape solids during winemaking [96]. The majority phenolic fraction in red wine is constituted by flavonoids (>85%), especially procyanidins (i.e., flavan-3-ol oligo/polymers; condensed tannins). Actually, red wine is one of the richest dietary sources of procyanidins [97], a type of compound recognized to possess a range of biological activities, and that is related with the disease preventive properties of plant-based diets [98,99]. Red wine is also rich in anthocyanins (especially young red wine) and flavonols, with acknowledged biological activities, including antioxidant, anti-inflammatory, antiproliferative, or gene modulating abilities [100], which are also considered to contribute to the health protective effects of fruits and vegetables. Hydroxycinnamic acids and their tartaric esters are the most important phenolic compounds in white wine, while other phenolics, like hydroxybenzoic acids, stilbenes (e.g., resveratrol), lignans, or dihydroflavonols are usually present in low concentrations in either type of wine, usually not exceeding a few mg/L [96].

Polyphenols, and especially flavonoids, have been proposed to be the main vasoactive components in red wine. They have been reported to be able to modulate the plasmatic lipid profile to a healthy shape, reducing triglyceride and LDL-cholesterol circulating levels [90,101,102]. They may also improve both systolic and diastolic blood pressure, stimulate endothelial-dependent vasodilation by enhancing nitric oxide (NO) generation, decrease platelet aggregation, and inhibit the activity of inflammatory enzymes and the production of several types of proinflammatory and oxidant mediators [103,104,105]. Many recent reports have been published dealing with the putative health effects of polyphenols, either from wine or other plant sources, and their possible mechanisms of action (see, e.g., [17,106,107,108,109,110,111]), and thus it does not seem necessary to insist herein. 

In addition to polyphenols, other bioactive phenolic and non-phenolic components can also be present in wine that might contribute to the putative health effects and that are usually less considered. Thus, during must fermentation, yeasts catabolize aromatic amino acids—such as tyrosine, tryptophan, and phenylalanine—to their respective aromatic alcohols, tyrosol, tryptophol, and phenyl ethanol, which also possess bioactive properties and are also associated with some of the beneficial effects of moderate wine consumption [112]. Tyrosol has been indicated to be the second most abundant non-hydroxycinnamate phenolic in many wines, with concentrations that may reach up to 95 mg/L. Its antioxidant and anti-inflammatory properties were suggested to contribute to the beneficial effects attributed to a moderate consumption of wine [113,114]. Among others, tyrosol was found to be able to inhibit the LPS-induced production of pro-inflammatory cytokines tumor necrosis, like factor alpha (TNF-α), and interleukins IL-1β and IL-6 in human peripheral blood mononuclear cells at nanomolar concentrations, either alone or in synergy with caffeic acid [113,114]. Hydroxytyrosol is also present in wine in levels under 10 mg/L [115,116,117], but it can also be formed in the human organism from hydroxylation of tyrosol. De la Torre et al. [118] found that the consumption of moderate doses of wine or olive oil by healthy subjects led to a higher increase in urinary concentrations of hydroxytyrosol in the wine group, despite the fact that the amount of hydroxytyrosol administered was fivefold greater in the olive oil group (1.7 mg vs. 0.35 mg). This was explained by the biotransformation of tyrosol to hydroxytyrosol; besides, the alcohol could help to increase the bioavailability of the tyrosol present in the wine. The authors indicated that a single glass of wine was at least equivalent to 25 mL (22 g) of virgin olive oil in its capacity to increase hydroxytyrosol concentrations in the body, leading to similar beneficial effects. The same group found that there was a direct association between wine consumption and the urinary concentrations of tyrosol and hydroxytyrosol determined in individuals at cardiovascular risk included in the PREDIMED study [119], suggesting that the endogenous formation of hydroxytyrosol might explain part of the cardiovascular benefits associated with light-to-moderate wine consumption.

Another bioactive compound that may contribute to the health benefits of wine is melatonin (*n*-acetyl-5-methoxytryptamine). This is a neurohormone secreted from the pineal gland, with well-characterized antioxidant, anti-inflammatory, and immune-modulating properties. It also contributes to the regulation of the circadian rhythms and has been attributed to tumor inhibitory activities and positive effects on the cardiovascular system, lipid, glucose metabolism, and neuroprotection [120,121]. It is present in grapes and can also be formed in wine from tryptophan metabolism by yeasts [122]. Actually, its content in wine is mostly influenced by the fermentation process, where the yeast strain and the fermentation time are the most influential factors [122]. It has been shown that blood levels of melatonin and total antioxidant capacity in plasma increased after the dietary intake of food containing it [123,124,125]. Melatonin concentrations ranging from a few µg/L to more than 150 µg/L have been reported in wine [126], which is higher than those found in most fruits and vegetables. Moreover, most fruits and vegetables are usually situated in the low ng/g level, with only a few products, such as mushrooms, coffee beans, or some berries showing contents in the µg/g range [120]. Therefore, wine can be considered a significant source of dietary melatonin, though it is not unlikely that it could be a contributor to the beneficial effects associated with wine consumption [121].

## 5. What about Resveratrol?

A phenolic compound that has been frequently associated with the putative beneficial effects of wine is the stilbene resveratrol (3,4′,5-trihydroxy-trans-stilbene), a phytoalexin that can be found in grape skin and is extracted into wine during winemaking. The average contents of resveratrol in wine does not usually exceed a few mg/L [96]. Since white wine is not usually submitted to maceration with grape solids, it possesses lower resveratrol concentrations than red wine.

Dietary sources of resveratrol are scarce and, in addition to grapes, they include rhubarb, peanuts, or berries, though they are always present in low levels. Actually, grapes and wine are considered the most relevant food sources for humans [127]. Stilbenes are synthetized by plants in response to biotic or abiotic stress, so that exposure to UV radiation can induce the formation of resveratrol in grapes, increasing its concentration by up to tenfold [128]. Post-harvest UV irradiation has been employed as a strategy to increase resveratrol levels so as to “functionalize” grapes [129].

The presence of resveratrol in wine was firstly described in 1992 [130], suggesting that it might be an active component in the lowering effects of serum lipids associated with wine consumption. Since then, a high number of studies have been published reporting a diversity of bioactivities and multiple potential health outcomes for stilbene, including antioxidant, anti-inflammatory, anti-obesity, chemopreventive, glucose-modulating, cardiovascular protective, or calorie restriction mimicking effects [131]. A departure point in resveratrol research could be established in the study by Jang et al. [132], reporting its ability to inhibit the enzymatic activity of both forms of cyclooxygenase (COX1 and COX2), suggesting that it may behave as an anti-inflammatory and anticarcinogenic agent. Further studies showed that it was able to enhance stress resistance and extend lifespan in various model organisms, including *Saccharomyces cerevisiae*, *Caenorhabditis elegans*, *Drosophila melanogaster*, fish, and mice [133,134,135]. Those effects were related to the activation of Sir2 proteins (sirtuins), a family of NAD^+^-dependent deacetylases and mono-ADP-ribosyltransferases involved in key regulation processes, such as glucose and insulin production, fat metabolism, the regulation of the p53 tumour suppressor, and cell survival [136]. 

Later on, several authors have also explored the effects of resveratrol on obesity, brain function, and visual performance. The results obtained in a number of studies in cell, animal, and human trials revealed that resveratrol and related stilbenes were able to inhibit adipocyte differentiation and proliferation, decrease lipogenesis, and promote lipolysis and fatty acid beta-oxidation [137], pointing out that it may be used as an anti-obesity agent. Regarding brain function, Kennedy et al. [138] found that the oral administration of a single dose of resveratrol (250 or 500 mg) to healthy adults increased cerebral blood flow during task performance in a dose-dependent way without affecting cognitive function. Furthermore, Evans et al. [139] reported that daily consumption of 150 mg of resveratrol for 14 weeks enhanced verbal memory and overall cognitive performance in postmenopausal women. Another study in postmenopausal women concluded that supplementation with 75 mg of trans-resveratrol twice a day for a year improved overall cognitive performance and cerebrovascular responsiveness to cognitive stimuli, which was also associated with a reduction of fasting blood glucose [140]. By contrast, a nutritional intervention with 200 mg/day of resveratrol failed to show significant improvements in verbal memory after 26 weeks in healthy elderly individuals [141]. Moreover, in a meta-analysis on the results obtained from four randomized clinical trials, Farzaei et al. [142] did not conclude significant effects on memory and cognitive performance assessed by auditory verbal learning test. Similarly, Marx et al. [143] concluded that, despite the fact that resveratrol supplementation might improve cognitive performance, the results obtained among clinical trials are limited and inconsistent. As for visual performance, studies carried out in different retinal cell lines found that resveratrol at micromolar concentrations was able to protect them from damage caused by oxidative stress and hyperglycemia-induced low-grade inflammation, suggesting that it might contribute to preventing age-related ocular disorders like cataracts, glaucoma, or macular degeneration [144,145,146]. Additionally, oral administration of resveratrol (5 to 200 mg/kg for 5 days) to mice was seen to prevent endotoxin-induced uveitis by inhibiting oxidative damage, leading authors to propose that supplementation with resveratrol is a possible strategy to treat ocular inflammation [147].

However, despite the range of evidence on the potential benefits of resveratrol obtained in model and preclinical studies, attempts have failed to come to clear and consistent outcomes in cohort and clinical trials [148,149]. It must also be highlighted that the available studies have been performed using relatively high doses of resveratrol, which are unlikely to be provided by the diet when taking into account the scarcity of food sources and the very low concentrations at which stilbenes are present. It does not seem that Mediterranean diets, either with or without wine, can represent further improvements in this sense. Thus, it should not be expected that resveratrol may have a relevant contribution to the beneficial health effects associated with Mediterranean diets or any other type of diet. Supplementation or therapeutical approaches might, therefore, be the way to take advantage of its potential benefits. Nonetheless, much work seems still required in this respect. As recently reviewed by Ren et al. [148], poor pharmacokinetics and low potency—as well as possible toxicity issues, including gastrointestinal disorders, headache, rash, or nephrotoxicity [131,148]—seem the main bottlenecks to overcome for its nutritional or therapeutical application. The development of more potent analogues and/or novel resveratrol formulations to enhance its bioavailability may be promising strategies to take it from bench to people [148].

## 6. The Social Context

Polyphenols are not the only reason that has been argued to support the beneficial effects associated with wine consumption, but socioeconomical and contextual factors also matter and could be even more important. Indeed, when interpreting the relationship between wine consumption and health, the underlying lifestyle and dietary patterns have to be considered, as they can be as influential on the health outcome as the type of drink. Mediterranean diets are themselves considered to constitute healthy dietary and lifestyle behaviour, making it difficult to extract the contribution of wine; otherwise, they might counteract the negative impact of alcohol on the organism.

It has been claimed that the MedDiet involves a “Mediterranean way of drinking”, that is, a regular, moderate wine intake mainly consumed with meals [6]. When consumed with meals, wine tends to be sipped more slowly as compared to other alcoholic drinks, which may provide metabolic advantages. Among others, the concomitant presence of food in the stomach slows down gastric emptying and subsequent ethanol absorption which favours hepatic metabolism and clearance, lowering the peak of alcohol concentration in the blood [150]. It has been reported that, when consumed within meals, alcohol intake is associated with a lower risk of acute myocardial infarctions [151]. The concurrent presence of food might also reduce the amount of alcohol available to the oral microbiota, which has the capacity to metabolize ethanol to acetaldehyde, a compound associated with the tumorigenic effects of ethanol in the upper gastrointestinal tract [152]. It has also been observed that when wine is consumed with food, the onset of the plasma uric acid elevation coincides with the period of postprandial oxidative stress produced after a meal, which may contribute to the wine’s protective effects [153]. Moreover, the presence of alcohol may improve the bioavailability of polyphenols in the food bolus, which would thus be more easily assimilated [154]. 

Studies in countries where wine is not the traditional alcoholic drink have also supported that a preference for wine is associated with healthy outcomes and more favourable dietary patterns [155]. Burke et al. [156], in a health screening on middle-aged men in Australia, found that a preference for wine was related to a greater consumption of fruit, vegetables, and bread, as compared to people that preferred beer. In a survey in Finland, Mannisto et al. [157] observed that wine drinkers had significantly higher intakes of antioxidants in their diet, indicating a greater consumption of fruit and vegetables than groups with other drink preferences. A higher intake of fruits, salads, cooked vegetables, fish, and olive oil was also found by Tjonneland et al. [158] in those that preferred wine, as compared with other alcoholic drinks, in a cross-sectional study conducted in Denmark. Similarly, Sluik et al. [159], in a representative sample of people from the Netherlands, found that wine drinkers consumed less energy and more vegetables and fruit juices, while the choice of beer was associated with a higher intake of meat, soft drinks, margarine, and snacks. All those behaviours associated with wine choice result in diets closer to the MedDiet, supporting the idea that it is not only wine, but the associated dietary and lifestyle patterns which contribute to healthier outcomes. Interestingly, this type of association has not been found in studies performed in some Mediterranean countries, as was the case of some Italian [160] or Spanish cohorts [161,162], where no significant correlation between wine consumption and healthier dietary habits was observed in relation to non-drinkers or consumers of other alcoholic beverages.

## 7. Risks of Wine Consumption

Despite the fact that moderate consumption may have health benefits, it is also well-known that alcohol, even at a low level of consumption, has some risks. It is well-known that there is a causal relationship between alcohol intake and the incidence of a variety of pathologies—particularly liver diseases—which, in their more severe form, such as the alcoholic hepatitis, lead to a mortality rate exceeding 50% in three months. Other manifestations, like steatosis of alcoholic cirrhosis, are initially less severe, although in advanced cirrhosis, the median of survival is situated around 1–2 years [163]. In addition, there are also well-established relationships between alcohol intake and incidence of pancreatitis and diverse types of cancer, as well as some infectious diseases and non-intentional injuries.

A comprehensive report on alcohol-attributable deaths was released in 2018 within the frame of the Global Burden of Diseases, Injuries, and Risk Factors Study 2016 [164]. For that, a meta-analysis of relative risks for 23 health outcomes associated with alcohol use was made using 694 data sources of individual and population-level alcohol consumption, along with 592 prospective and retrospective studies on the risk of alcohol use. The study used 195 locations and a time span from 1990 to 2016, including people aged above 15 of both sexes. It was concluded that alcohol use was a leading risk factor for the global disease burden worldwide, causing substantial health loss from many causes and accounting for nearly 10% of global deaths among people aged 15–49 years. The risk of all-cause mortality, and of cancers specifically, raised with increasing levels of consumption, with no level of consumption that can be considered free of risks [164].

The International Agency for Research on Cancer (IARC) classifies alcohol as a Group 1 carcinogen, causally associated with the development of cancers of the upper digestive tract and liver, and has sufficient evidence to be positively associated with colorectum and female breast cancer, without differences among the type of alcoholic drink [165]. The existence of a high association between alcohol intake and the increased risk of different types of cancers was confirmed in many prospective studies. In a meta-analysis carried out on 156 epidemiological studies, Corrao et al. [166] concluded that the risk for all types of cancer significantly increased for ethanol intakes of above 25 g/day. Also, a highly significant association was found for liver cirrhosis and essential hypertension, although, for coronary heart disease and ischemic stroke, a reduction in the risk was observed with a minimum consumption of 20 g alcohol/day [66]. 

An aspect to notice is that observational studies on alcohol and health usually consider average alcohol consumption, which may hide risky drinking behaviours, such as irregular binge drinking, that always involves higher health risks and mortality rates [167,168]. A pattern of irregular heavy drinking is associated with pathophysiological mechanisms that increase the risk of sudden cardiac death, hypertension, atrial or ventricular fibrillation, and cardiomyopathy, even if the average consumption is comparable to moderate consumption [169]. Heavy drinking during pregnancy is known to produce foetal alcohol syndrome, leading to abnormalities and mental retardation. Nevertheless, there is also evidence that prenatal exposure to light to moderate levels of alcohol could affect foetal development and result in decreased body weight, neurodevelopmental deficits, and long-term effects on the growth of children [170,171]. All in all, there is no level of alcohol that can be considered safe during pregnancy. As a result, its consumption must be avoided by pregnant women, as well as during the period of breastfeeding. Similarly, alcohol must be avoided by younger people: adolescent alcohol use shows clear positive relationships with total mortality and is associated with an increased risk for development of chronic alcohol use disorders in adulthood [172]. 

It has been suggested that the detrimental effects of ethanol might be partly counterbalanced by the polyphenols contained in wine and other foods that play a part in the MedDiet, like extra-virgin olive oil [173], although this may be more of a perception that is not supported by consistent studies in humans. In a recent position paper on Dietary Guidelines for the Spanish Population [174], the Spanish Society of Community Nutrition (SENC) established that the consumption of alcoholic beverages is not encouraged or recommended in any case. Nevertheless, taking into account the prevalence of the Mediterranean uses and customs in Spain, an optional consumption of wine in limited amounts (no more than 40 g alcohol/day for men and no more than 20 g alcohol/day for women) and with meals is suggested only for adults who so desire and are not subject to contraindication due to a health condition or medication use. The SENC also highlighted that people who do not use alcoholic beverages should not start drinking because of its potential beneficial effects, and that equivalent results can be achieved through an adequate diet without the potential risks of alcohol.

## 8. Concluding Remarks

The Mediterranean diet has been associated with beneficial health outcomes in the prevention of chronic degenerative disorders, including cardiovascular diseases, type-2 diabetes, cognitive decline, or cancers. Its benefits were recognized by the UNESCO, which in 2010 inscribed the Mediterranean diet as an Intangible Cultural Heritage of Humanity. A feature of the MedDiet that has been related to its health benefits is that it contributes significant amounts of antioxidants, and especially polyphenols, whose regular intake is related to beneficial effects on the lipids profile, blood pressure, glucose metabolism, adiposity, and inflammatory processes. Virgin olive oil and moderate wine consumption have been indicated as two distinctive hallmarks of the MedDiet, contributing to its health benefits [44]. Indeed, olive oil represents a differential Mediterranean product, with a peculiar phenolic composition based on secoiridoids and derived phenolic alcohols, described to be able to improve the blood lipidic profile, maintain blood pressure, and provide anti-inflammatory properties, as recognized by the EFSA with a health claim.

On the other hand, a moderate consumption of wine, especially red wine, has been proposed to provide some degree of protection against cardiovascular diseases, diabetes mellitus, or cognitive decline, which has been related to its polyphenol content. The available studies in this respect are, however, limited by their observational nature, and there is a lack of randomized clinical trials that may prove a causal relationship. Furthermore, wine contains alcohol, which even at moderate consumption increases the risks of liver disorders and several types of cancers, among other diseases, Although the Mediterranean habit of drinking wine with meals may delay ethanol absorption and favour its more rapid clearance, at the same time that it may contribute to a decrease in postprandial oxidative stress produced after a meal. Furthermore, although polyphenols present in wine are also found in fruits and vegetables that lack the risks associated with alcohol, the concomitant presence of ethanol in the food bolus might make wine polyphenols more bioavailable. Some authors have, however, highlighted that a high wine and total alcohol intake, particularly by men, can represent a problematic aspect of the Mediterranean diet that may have not been critically evaluated [175]. Indeed, the potential risks of wine consumption, even at moderate doses, may have been overlooked or undervalued by many authors, which inadvertently may have disclosed a confusing message, although not only restricted to the context of the Mediterranean diets. Certainly, it does not seem wise to think of wine or any other alcoholic drink as an element for health promotion, but the risks of alcohol should always be considered in the first place. Releasing any message that might induce people to drink in the hope of gaining health benefits could likely have more harmful than beneficial consequences.

All in all, it is not easy to give a simple answer to the question of whether wine should be considered a key food contributing to the beneficial health outcomes of the MedDiet. Despite the fact that it is excluded from the diet in many Mediterranean areas for religious reasons, we do think that it definitely constitutes a distinguishing feature of many Mediterranean cultures, and plays an undeniable part of their historical legacy. In those regions, wine can be a relevant contributor to polyphenol intake and could be considered a side element in the beneficial health effects of the MedDiet, provided that it is consumed in the ‘traditional’ way, that is, light to moderate regular consumption with meals. In our opinion, wine has to be regarded as a fruitive food, to be enjoyed responsibly and in moderation, in a convivial environment and in the context of an adequate diet. In this case, it may constitute another element of a healthy lifestyle, provided that there are no reasons that advise against their intake.

## Figures and Tables

**Table 1:** Main bioactive compounds provided by representative foodstuffs of the Mediterranean diets.

Food Item	Main Bioactive Compounds
Fresh fruit	Vitamin C, polyphenols, dietary fiber
Citrus fruits	Vitamin C, flavonoids
Nuts	Polyunsaturated fatty acids, phytosterols, vitamin E
Whole grains	Complex carbohydrates, dietary fiber
Legumes	Proteins, dietary fiber, saponins
Raw vegetables (tomatoes, carrots)	Hydrosoluble vitamins, carotenoids
Leafy green vegetables	Folic acid, dietary fiber
Cruciferous	Glucosinolates
Fish	n-3 Long-chain polyunsaturated fatty acids, high-quality proteins
Dairy products	Calcium, bioactive peptides, high-quality proteins
Eggs and poultry	High-quality proteins
Extra virgin olive oil	Monounsaturated fatty acids, polyphenols, phytosterols
Red wine	Polyphenols
Allium compounds	Sulphur compounds

## Sample Availability

Samples of the compounds are not available from the authors.
